# Hordenine Activated Dermal Papilla Cells and Promoted Hair Regrowth by Activating Wnt Signaling Pathway

**DOI:** 10.3390/nu15030694

**Published:** 2023-01-30

**Authors:** Caibing Wang, Kai Zang, Zexin Tang, Ting Yang, Xiyun Ye, Yongyan Dang

**Affiliations:** 1 Institute of Biomedical Sciences, East China Normal University, Shanghai 200241, China; 2 Shanghai Key Laboratory of Regulatory Biology, School of Life Science, East China Normal University, Shanghai 200241, China

**Keywords:** hordenine, DPCs, hair growth, Wnt/β-catenin signaling pathway

## Abstract

Hordenine is effective in treating hyperpigmentation, fighting diabetes and resisting fibrosis and acute inflammation. However, the role of Hordenine on hair growth has not been elucidated. Here, we found that Hordenine treatments significantly enhance proliferation of primary mouse dermal-papilla cells (DPCs) and increase the activity of DPCs in a dose-dependent manner. Additionally, Hordenine markedly promoted the elongation of the hair shaft in the model of in vitro-cultured mouse vibrissa follicle and accelerated hair regrowth in a mouse model of depilation-induced hair regeneration. Real-time PCR, Western Blot and immunofluorescent assays showed that nuclear β-catenin and its downstream gene expression such as Lef1, Axin2, Cyclin D1 and ALP were greatly upregulated in DPCs and mouse hair follicles after Hordenine treatments. Moreover, the increased DPCs’ proliferation and hair shaft elongation of cultured mouse vibrissa follicles induced by Hordenine treatments were rescued by a Wnt/β-catenin signaling inhibitor, FH535. These data indicate that Hordenine can effectively enhance DPCs’ activity and accelerate hair regrowth through activating the Wnt/β-catenin signaling pathway. Therefore, these findings suggest Hordenine/its derivatives may be potentially used for preventing and treating alopecia in the future.

## 1. Introduction

Hair follicles are highly specialized organs with long-lasting growth and regenerative capabilities [1]. Each hair follicle repeatedly undergoes a growth cycle that comprises three distinct phases: anagen, catagen and telogen [2]. When the hair growth cycle is disrupted, alopecia and hair thinning may occur [3]. Currently, alopecia has become a common worldwide problem that affects many people at some points in their lives [4]. However, few drugs have been approved to treat hair thinning or alopecia due to a lack of efficiency or the side-effect profiles. Thus, it is urgent to find the new and safe compounds that can prevent and treat alopecia in the future.

Now, natural plant products have been used in treating different health-related diseases because of the merit of few side effects. Hordenine, also known as p-dimethyl aminoethyl phenol, is a kind of phenethylamine alkaloid which is a natural ingredient in many plants, such as cacti [5] and germinated barley seeds [6]. Recent studies reported that Hordenine could inhibit hyperpigmentation [7], fight diabetes and fibrosis [8] and have an anti-inflammatory capacity [9]. Hordenine can also be used as a potential pyruvate dehydrogenase kinase 3 (PDK3) inhibitor of great value in the treatment of lung cancer [10]. In addition, Hordenine was demonstrated to affect the function of the nervous system, such as inhibiting monoamine oxidase B and the uptake of norepinephrine [11]. However, it is still unclear if Hordenine can affect hair health and hair growth. We fortunately found the potential of Hordenine in relation to hair growth through screening the compound library in this study.

Hair growth and regeneration are regulated by a variety of signaling pathways. Among these, the Wnt/β-catenin signaling pathway is one of the most important signaling transduction pathways in regulating the growth, development and cycle of hair follicles [12,13]. The activation of the Wnt/β-catenin signaling pathway can promote the proliferation and migration of hair follicle cells, including hair follicle stem-cells, hair matrix cells and dermal papilla cells [14,15], thereby inducing the transition of hair follicles from telogen to anagen. In addition, Wnt signaling can promote angiogenesis and provide a nutrient environment for hair follicle growth [16,17]. Therefore, Wnt/β-catenin signaling can be used as a potential target for the prevention and treatment of alopecia. However, there is still a lack of effective and safe modulators for the Wnt signaling pathway.

In this study, we found that Hordenine has a new function of promoting the proliferation of DPCs and enhancing the activities of DPCs. By using in vitro-cultured mouse vibrissa-follicle model and in vivo mouse depilation-induced hair regeneration model, we demonstrated that Hordenine can effectively promote the elongation of the hair shaft, facilitate anagen entry and accelerate hair growth by activating the Wnt/β-catenin signaling pathway. These data highlight a novel role for Hordenine in the regulation of HF growth and provide a potential strategy for the treatment of alopecia.

## 2. Methods and Materials

### 2.1. Isolation and Culture of Mouse Vibrissa Hair Follicles and Primary DPCs

The 7-week-old female C57BL/6 mice were obtained from the SPF Laboratory Animal Center of the East China Normal University. The animal experiment in this study was approved by the Experimental Animal Ethics Committee of East China Normal University, and the approval number is M20181103. After anesthesia with pentobarbital sodium, the mouse skin of the vibrissa was taken to isolate hair follicles with tweezers. The isolated mouse vibrissa hair follicles were then cultured in William’s Medium E medium (Gibco, 12551032, New York, NY, USA). Then, the hair bulb was cut from the lower end of the hair follicle, digested with 0.2% collagenase I (Gibco, 17100017, New York, NY, USA) for 1 h, and filtered with a 40 μm filter to remove the cells. The remaining tissues were transferred to a 60mm petri dish containing 5 mL of DMEM medium with 15% fetal bovine serum (FBS) (Gibco, New York, NY, USA) and 1% antibiotic-antimycotic solution (Gibco, New York, NY, USA) for a week to obtain a large number of DPCs.

### 2.2. Cell Viability Assay

DPCs were seeded in 96-well plates with 1 × 10^4^ cells per well. When the confluence reached 80%, cells were treated with Hordenine of different concentrations for 48 h. Then, the medium was removed and replaced with 120 μL pre-mixed medium containing 100 μL fresh medium and 20 μL MTS (Promega, G5421, Madison, WI, USA) for each well. After incubation at 37 °C for 0.5 h, the absorbance was measured at 490 nm using a SPECTRA MAX 190 spectrometer (Molecular Devices, San Jose, CA, USA).

### 2.3. Clone Formation Assay

The DPCs were seeded into a 6-well plate with 500 cells per well. After culturing for 24 h, the DPCs were treated with Hordenine of 0, 25 and 50 µmol/L and cultured for 10 consecutive days. The medium was changed every 2 days. When there were visible clones with 50-150 cells per clone in the culture dish, the plate was treated with 4% paraformaldehyde for 15 min, and dyed with 1 mL of Crystal Violet Staining Solution (Beyotime, C0121, Shanghai, China) for 20 min in room temperature. Then, the plate was washed, air-dried, and photographed with a digital camera (Nikon, Tokyo, Japan).

### 2.4. Immunofluorescence Staining

DPCs were seeded in a 24-well plate at a density of 3 × 10^4^ and cultured for 24 h. Then, Hordenine at the concentration of 0, 25 and 50 µmol/L was added to treat DPCs for 24 h. Then, cells were washed with PBS for three times, fixed in 4% paraformaldehyde for 15 min, permeabilized with 0.1% Triton X-100, and blocked with 1% FBS for 0.5 h. The cells were then incubated overnight at 4 °C with the specific primary antibodies, followed by incubation for 2 h with fluorescent secondary antibody and staining with DAPI for 5 min. Photographing were conducted under an Olympus microscope. All the primary antibodies used in immunofluorescence staining were as follows: 1:200 anti-Ki67 antibody (Abcam, 16667, Cambridge, UK), 1:200 anti-β-catenin antibody (Proteintech, 51067-2-AP, Chicago, IL, USA), 1:100 anti-ALP antibody (Abcam, 65834, Cambridge, UK), 1:100 anti-Versican antibody (Abcam, 19345, Cambridge, UK).

### 2.5. EDU Cell Proliferation Assay

The primary DPCs were inoculated in a 24-well plate in triplicate, incubated for 24 h, then treated with Hordenine for 24 h. The EDU assay was carried out according to the manufacturer’s instructions (BeyoClick™ EdU Cell Proliferation Kit with Alexa Fluor 488, C0071S, Beyotime, Shanghai, China). Cultures were observed under a fluorescence microscope (Olympus, Tokyo, Japan).

### 2.6. Real-Time Quantitative PCR

DPCs were seeded into a 6-well plate with 2.5 × 10^5^ cells per well. After treating the cells with 0, 25 and 50 µmol/L Hordenine for 24 h, the total RNA was extracted with Magzol Reagent (Magen, KD210300, Guangzhou, China). Reverse-transcription PCR was conducted with 1000 ng of total RNA using a Hifair^®^ Ⅱ 1st Strand cDNA Synthesis SuperMix (Yeasen, 11120ES60, Shanghai, China). Q-PCR was performed with Hieff^®^ qPCR SYBR Green Master Mix (Yeasen, 11202ES03, Shanghai, China) to detect the expression of related genes according to the following conditions: 95 °C for 15 s, 55 °C for 30 s, and 72 °C for 30 s (40 cycles). β-actin was used as an internal control.

All the primers used in the real-time quantitative PCR were as follows: *ALP*, 5′-CCAACTCTTTTGTGCCAGAGA-3′ (forward) and 5′-GGCTACATTGGTGTTGAGCTTTT-3′ (reverse); *Versican*, 5′-TTTTACCCGAGTTACCAGACTCA-3′ (forward) and 5′-GGAGTAGTTGTTACATCCGTTGC-3′ (reverse); *Wnt3a*, 5′-CTCCTCTCGGATACCTCTTAGTG-3′ (forward) and 5′-GCATGATCTCCACGTAGTTCCTG-3′ (reverse); *β-catenin*, 5′-ATGGAGCCGGACAGAAAAGC-3′ (forward) and 5′CTTGCCACTCAGGGAAGGA-3′ (reverse); *Lef-1*, 5′-AGAAATGAGAGCGAATGTCGTAG-3′ (forward) and 5′-CTTTGCACGTTGGGAAGGA-3′ (reverse); *Axin2*, 5′-TGACTCTCCTTCCAGATCCCA-3′ (forward) and 5′-TGCCCACACTAGGCTGACA-3′ (reverse); *Cyclin d1*, 5′-GCGTACCCTGACACCAATCTC-3′ (forward) and 5′-CTCCTCTTCGCACTTCTGCTC-3′ (reverse).

### 2.7. Western Blot

The DPCs were seeded in a 6-well plate and treated with 0, 25 and 50 µmol/L Hordenine for 24 h. The total cell protein was extracted with RIPA lysis buffer (25 mM Tris pH 7.6, 150 mM NaCl, 1% NP-40, 0.1% SDS, 1.0% Triton X-100, 1% Deoxycholate 5 mM EDTA Add protease/phosphatase inhibitors). After separating the protein by SDS-PAGE, the protein was transferred to the nitrocellulose membrane (NC membrane). After blocking with 5% skim milk for 1 h, the membranes were incubated with the primary antibodies overnight on a shaker at 4 °C. The blots were washed and incubated with the secondary antibody conjugated with fluorescent monomer for 2 h. Membranes were visualized using an LI-COR Odyssey infrared imaging system (LI-COR, Lincoln, NE, USA). All the primary antibodies used in Western blot were as follows: 1:1000 anti-ALP antibody (Abcam, 65834, Cambridge, UK), 1:1000 anti-β-catenin antibody (Proteintech, 51067-2-AP, Chicago, IL, USA), 1:1000 anti-P-GSK3β antibody (CST, 5558, Danvers, IL, USA), 1:1000 anti-Lef-1 antibody (Proteintech, 14972-1-AP, Chicago, IL, USA), 1:1000 anti-Axin 2 antibody (Abcam, 109307, Cambridge, UK), 1:1000 anti-Cyclin D1 antibody (Abcam, 16663, Cambridge, UK), 1:10,000 anti-GAPDH antibody (Proteintech, 60004-1-Ig, Chicago, IL, USA).

### 2.8. Animal Experiments

The 7-week-old C57BL/6 female mice were housed under a SPF condition (12-h light/dark cycle, 50% relative humidity, between 25 and 27 °C) with free access to food and tap water. All the mice were depilated for inducing synchronization of hair regeneration and then treated with Hordenine of 1 mmol/L and 2 mm/L for 25 days. The hair regrowth of the mice was recorded by taking pictures during this time. On the 6th day, mouse skin was taken for sectioning.

### 2.9. Hematoxylin and Eosin (HE) Staining

Skin samples were fixed in 4% PFA for 24 h, dehydrated in graded ethanol (50%, 75%, 85%, 95%, 100%), hyalinized by dimethylbenzene, embedded in paraffin and sectioned at 5-μm. The paraffin sections were rehydrated through xylene, graded ethanol series and stained with hematoxylin for 5 min and eosin for 30 s. Images were observed by an Olympus microscope (Olympus, Tokyo, Japan).

### 2.10. Data Analysis

The statistical significance was analyzed by GraphPad prism7 software. All quantitative data are expressed as mean ± standard deviation. All data were from the three independent experiments. The statistical significance of differences between the groups was determined by Student’s *t*-test. *p* < 0.05 was considered to be significant.

## 3. Results

### 3.1. Hordenine Has Little Toxicity to Epidermal and Dermal Cells

Hordenine, 4-(2-Dimethylaminoethyl) phenol, is a natural phenolic phytochemical compound extracted from germinated barley (Figure S1A (Supplementary Materials)) [7]. Firstly, we evaluated the cytotoxicity of Hordenine on the epidermal cell line HaCaT and the dermal cell line 3T3 cells by MTS analysis. As shown in Figure S1B,C, Hordenine with the concentration up to 200 μmol/L is not toxic to HaCaT and 3T3 cells. Even at the concentrations of 400 μmol/L and 800 μmol/L, 75% cells still maintain the activity comparable to the non-treated control. Thus, the results showed that Hordenine is safe for skin cells within the concentration of 200 μmol/L.

### 3.2. Hordenine Promotes Cell Proliferation and Elevates the Activity of Dermal Papilla Cells

**Figure 1:**
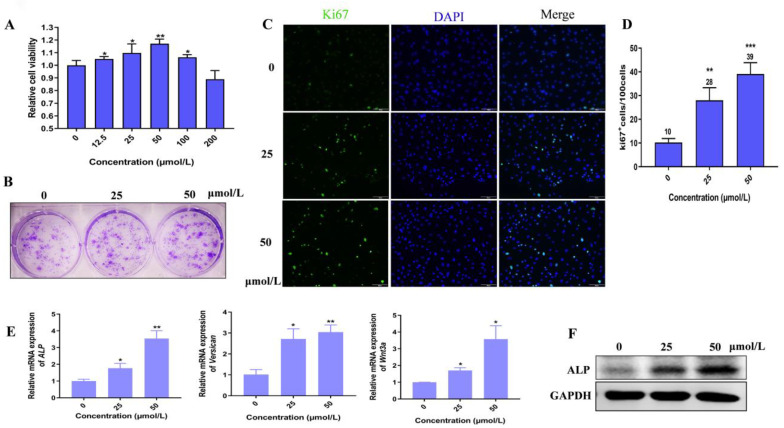
Hordenine enhanced the proliferation and activity of mouse dermal papilla cells. (**A**) Cell viability of DPCs treated with Hordenine in the concentrations of 0–200 μM/L for 48 h (*n* = 6). (**B**) Representative images of colonies formed by DPCs treated with Hordenine at 0, 25 or 50 μM/L. (**C**) Immunofluorescent staining for Ki67. The nuclei were counterstained by DAPI (blue). Scale bar, 100 μm. (**D**) The percentage of *Ki67*-positive DPCs, *n* = 5. (**E**) The mRNA expression of DPC’s activity markers (*ALP, Versican* and *Wnt3a*) by quantitative RT-PCR. (**F**) Western blot analysis of ALP in DPCs. Data are expressed as means ± SD. * *p* < 0.05, ** *p* < 0.01, *** *p* < 0.001, Hordenine versus control.

Considering dermal papilla cells (DPCs) are closely related to the induction of hair follicles’ (HFs) formation [18], we thus explored the effects of Hordenine on the cell viability of cultured primary DPCs from mouse vibrissa follicles. As shown in Figure 1A, Hordenine at the concentration of 200 μmol/L was also safe for DPCs. Moreover, the proliferation of DPCs was markedly increased by Hordenine at the concentration of 12.5–100 μmol/L, especially at 50 μmol/L. Consistently, the clone formation assay showed that Hordenine treatments significantly increased the colony counts of DPCs at the concentration of 50 μmol/L compared with the controls (Figure 1B). Immunofluorescence staining also exhibited a higher number of Ki67-positive cells in the Hordenine-treated groups than the controls (Figure 1C,D). Together, all the data suggest that Hordenine can promote the proliferation of DPCs.

Since alkaline phosphatase (ALP) is considered to be an indicator of DPCs’ induction potential [19], we further examined whether Hordenine affects the expression of ALP in DPCs. As expected, both the mRNA and the protein levels of ALP were markedly upregulated by Hordenine treatments in DPCs in a dose-dependent manner (Figure 1E,F). Previous studies reported that Versican was an extracellular matrix proteoglycan synthesized in the dermal papilla cells of anagen [20] and Wnt3a could maintain hair inductive activity [21]. Thus, we also detected the mRNA expression of Versican and Wnt3a in cultured DPCs. Consistently, our Q-PCR results showed that the mRNA levels of Versican and Wnt3a were also increased by Hordenine (Figure 1E). These results indicate that Hordenine can elevate the ability of proliferation and inductive activity of DPCs in a dose-dependent manner.

### 3.3. Hordenine Promotes the Growth of Hair Follicles In Vitro and Accelerates Anagen Entry in Depilatory Mice

**Figure 2:**
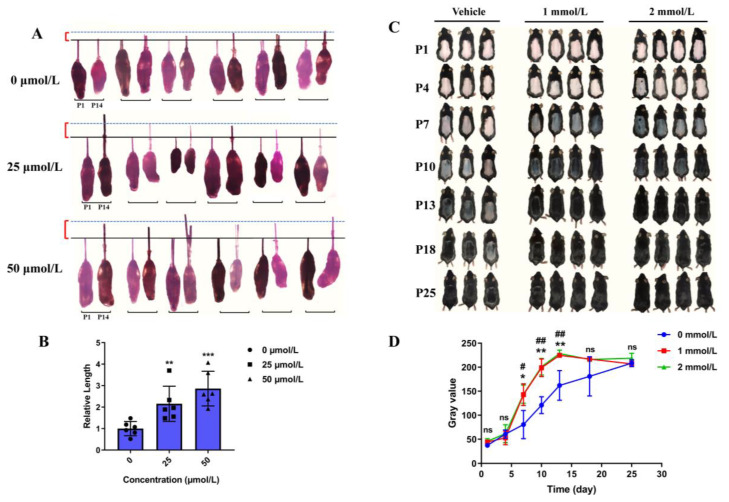
Hordenine promoted the elongation of hair shaft and accelerated hair regeneration of mice. (**A**) Mouse vibrissa follicles were isolated and cultured in William’s E with or without Hordenine for 14 days. Images of mouse vibrissa follicles were taken at day 1 and 14, respectively (*n* = 6). (**B**) The elongation length of mouse vibrissa follicles after Hordenine’s treatments for 14 days (*n* = 6). (**C**) Shaved dorsal skin of C57BL/6 mice was topically treated with vehicle or Hordenine (1 mmol/L or 2 mmol/L) for 25 days. Typical photos were taken to show hair regrowth of mouse dorsal hair. (**D**) The rate of hair regrowth was quantified by using the software of Image J. Data are expressed as means ± SD. * *p* < 0.05, ** *p* < 0.01, *** *p* < 0.001, 1 mmol Hordenine versus control; ^#^ *p* < 0.05, ^##^ *p* < 0.01, 2 mmol Hordenine versus control.

Considering the inductive properties of DPCs are required for HF regeneration and growth of the hair shaft, we established an in vitro model of cultured mouse vibrissa follicle and in vivo mouse model of depilation-induced hair regeneration to further detect the effects of Hordenine on hair growth. As shown in Figure 2A, Hordenine significantly increased the growth of the hair shaft in cultured mouse vibrissa follicles in a dose-dependent manner at day 14. The quantitative analysis showed the elongation length of hair shafts in Hordenine-treated group was significantly higher than that of the controls (Figure 2B). Meanwhile, after topically applying Hordenine (1 and 2 mmol/L) to the depilated backs of mice for 25 days, we observed that the Hordenine-treated mice entered into the anagen growth phase of the hair cycle faster than the vehicle-treated mice (Figure 2C). The gray values measured by image J software also supported that the rate of hair regeneration was significantly increased in both the 1 mmol/L and 2 mmol/L Hordenine-treated mice compared with the controls (Figure 2D). These results show that Hordenine can effectively promote hair growth in vitro and in vivo.

### 3.4. Hordenine Activates Wnt/β-Catenin Signaling Pathway In Vitro and In Vivo

**Figure 3:**
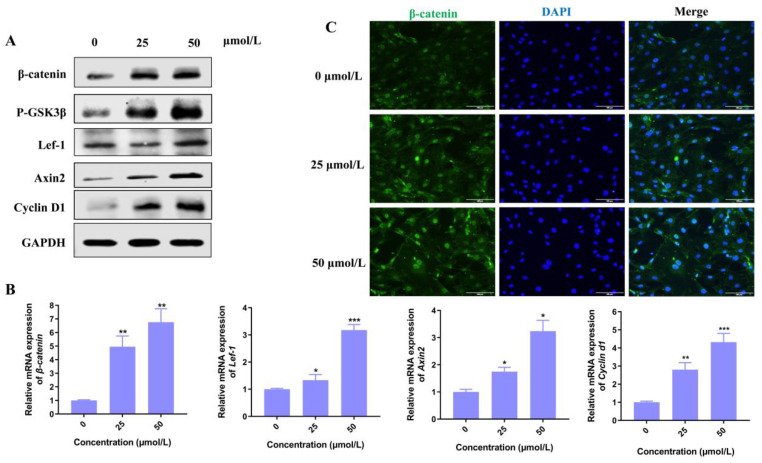
Hordenine activates Wnt/β-catenin signaling pathway in the mouse primary DPCs. (**A**) Western blot analysis of β-catenin, p-GSK-3β, Lef1, Axin2, Cyclin D1 in DPCs. (**B**) Analysis of the mRNA levels of β-catenin, Lef-1, Cyclin d1 and Axin2 in DPCs by quantitative RT-PCR. (**C**) Representative immunofluorescence images of β-catenin in DPCs treated with or without Hordenine for 24 h (Magnification, ×100; bars = 100 μm). Data are expressed as means ± SD. * *p* < 0.05, ** *p* < 0.01, *** *p* < 0.001, Hordenine versus control.

Among various signaling pathways, Wnt/β-catenin plays an essential role in hair morphogenesis and hair growth. Thus, we explore if the Wnt signaling pathway plays a key role in the promotion of hair growth induced by Hordenine. As shown in Figure 3A, the protein levels of p-GSK3β, β-catenin and its downstream molecules such as lef-1, Axin2 and Cyclin D1 were all significantly increased by Hordenine treatments. Consistently, Q-PCR results showed Hordenine treatments markedly upregulated the mRNA expression of β-catenin, Lef-1, Axin2 and Cyclin D1 in a dose-dependent manner (Figure 3B). It is known that β-catenin may translocate from the cytoplasm to the nucleus, where it might serve as a transcriptional factor to stimulate the expression of its downstream genes. Thus, we next detected the effects of Hordenine on the nuclear translocation of β-catenin in DPCs. Immunofluorescent data showed that Hordenine treatments greatly promoted the nuclear entrance of β-catenin in DPCs compared with the controls (Figure 3C). These results indicated that Hordenine treatments can activate the Wnt/signaling pathway in DPCs.

**Figure 4:**
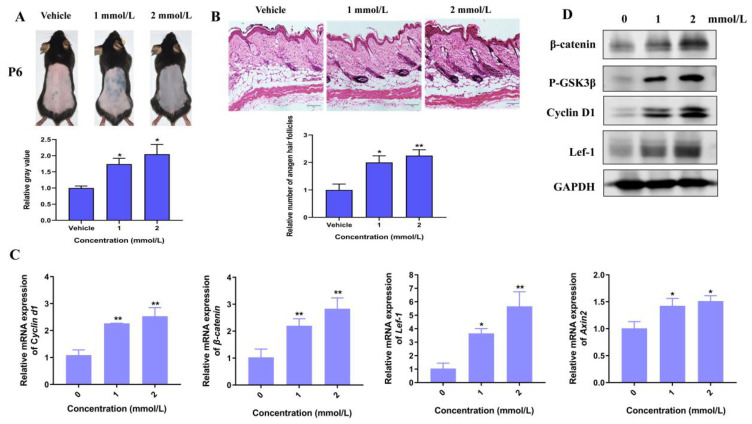
Hordenine increases the activity of Wnt/β-catenin signaling pathway in depilatory mice. (**A**) Representative photos of the mouse dorsal hairs in day 6 after Hordenine treatments. The bar chart showing the grey values of skin color. (**B**) Hematoxylin and eosin staining (HE) of the mouse dorsal skin hair follicles after Hordenine treatments in day 6 (Magnification, ×200; bars = 50 μm). The bar graph showing the average number of hair follicles in skin tissues. (**C**) The mRNA expression of *β-catenin*, *Lef-1*, *Cyclin d1* and *Axin2* were examined by RT-qPCR in mouse dorsal skin. (**D**) Western blot analysis of β-catenin, p-GSK-3β, Lef1, CyclinD1 in mouse dorsal skin. Data are expressed as means ± SD. * *p* < 0.05, ** *p* < 0.01, Hordenine versus control.

Moreover, we established a depilation-induced synchronizing hair regeneration model to confirm the effects of Hordenine on the Wnt/β-catenin signaling pathway. Comparing with the vehicle controls, the treatments of Hordenine at the concentration of 1 mmol/L and 2 mmol/L both markedly promoted hair follicles to enter a state of active growth at day 6 (Figure 4A,B). Importantly, the protein levels of β-catenin, p-GSK3β and Axin2 was markedly increased by Hordenine (Figure 4C,D). Meanwhile, Hordenine treatments greatly increased expression of the Wnt/β-catenin downstream target genes such as Lef-1 and Cyclin D1 at both the mRNA and protein levels (Figure 4C,D). These results suggest that Hordenine can activate the Wnt/β-catenin signaling pathway both in vitro and in vivo.

### 3.5. Hordenine Increased DPCs’ Proliferation and Hair Follicle Growth by Wnt/β-Catenin Signaling Pathway

**Figure 5:**
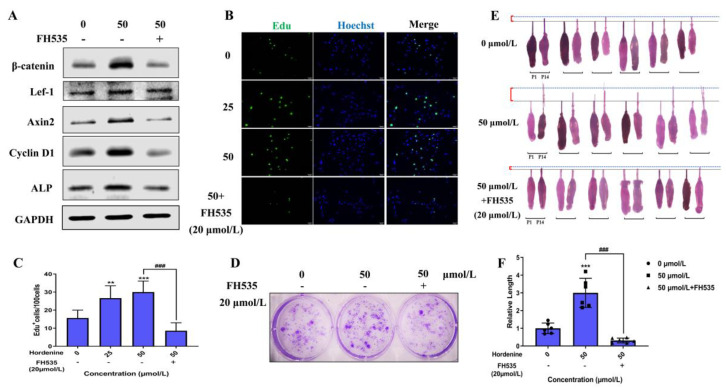
Hordenine increased DPCs’ proliferation and hair follicle regrowth by activating Wnt/β-catenin signaling pathway. (**A**) Western blot analysis of β-catenin, Lef1, Axin2, CyclinD1 and ALP in DPCs treated with Hordenine or co-treated with FH535. (**B**) Images of EdU staining in DPCs treated with or without Hordenine for 24 h. The nuclei were counterstained by DAPI (blue). Scale bar, 50 μm. (**C**) The percentage of EDU-positive DPCs, *n* = 5. (**D**) Representative images of colonies formed by DPCs treated with Hordenine or co-treated with FH535. (**E**) Mouse vibrissa follicles were isolated and cultured in William’s E with Hordenine in the presence or absence of FH535 (20 μmol/L) for 14 days. Indicate images of mouse vibrissa follicles at day 1 and 14, respectively (*n* = 6). (**F**) The elongation length of mouse vibrissa follicles after Hordenine’s treatment for 14 days (*n* = 6). Data are expressed as means ± SD. ** *p* < 0.01, *** *p* < 0.001, Hordenine versus control; ^###^ *p* < 0.001, Hordenine versus Hordenine plus FH535.

To further demonstrate if Hordenine plays its role by activating the Wnt/β-catenin signaling pathway, we used the Wnt inhibitor FH535 to treat DPCs and hair follicles. As shown in Figure 5A and Supplementary Figure S2A, FH535 could significantly downregulate the expression of β-catenin, Lef-1 and Axin2, exhibiting its effectiveness in inhibiting the Wnt/β-catenin signaling pathway. Critically, the results of the EdU-based cell proliferation assay showed that the number of EDU^+^ DPCs was markedly increased in the only Hordenine-treated group but greatly decreased with the addition of FH535 at 20 µmol/L (Figure 5B,C). The results of colony-forming assays also showed that the colony number was significantly increased upon treatment with Hordenine, which was reduced by FH535 administration (Figure 5D). Similar results were observed for the changes of protein levels of Cyclin D1, ALP and Versican using Western blot analysis and immunofluorescence staining (Figure 5A and Supplementary Figure S2B). Moreover, using an in vitro hair follicle culture model, we observed that Hordenine could effectively promote the growth of hair follicles, but co-treatment with FH535 and Hordenine significantly inhibited the Hordenine-induced hair growth (Figure 5E,F). All the data indicate that Hordenine increases the proliferation ability of DPCs, elevates the activity of DPCs and promotes hair growth depending on activating the Wnt signaling pathway.

## 4. Discussion

**Figure 6:**
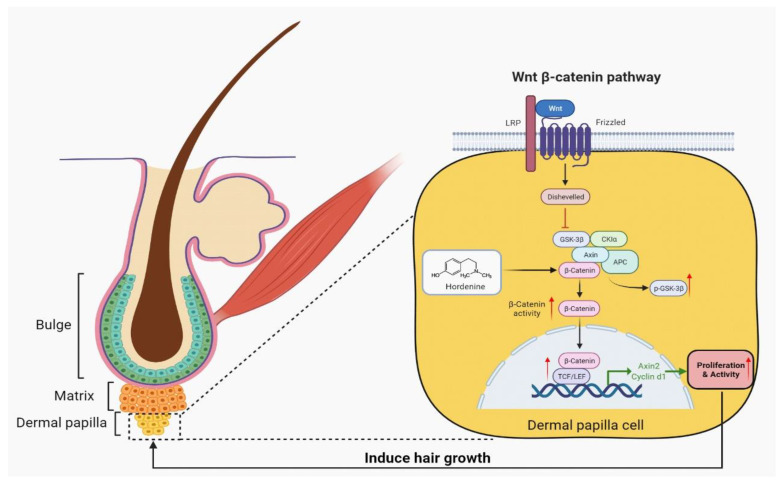
A model of promoting effects of Hordenine on hair regeneration. Hordenine significantly increased the proliferation of DPCs and enhanced their activity by activating Wnt/β-catenin signaling pathway, thus effectively promoting the elongation of hair shaft of mouse vibrissa follicles and accelerating hair regeneration in mouse dorsal skin.

Alopecia is a very common health problem with significant accompanying emotional distress [22]. Consequently, research into the development of therapeutic agents against alopecia is increasing. Currently, more and more attention has been focused on natural extracts from plants due to their lower toxicity [23]. However, natural compounds for treating alopecia in the clinic are still lacking. Therefore, the development of natural products is required including the exact mechanism underlying enhancement of hair growth and inhibition of alopecia. In this study, we are the first to discover that Hordenine can effectively increase the proliferation ability of DPCs and promote hair growth in vivo and in vitro by activating the Wnt/β-catenin signaling pathway (Figure 6). Thus, Hordenine, abundant in germinated barley [6], may be a potential candidate in accelerating hair growth and preventing alopecia in the future.

DPCs, which reside at the base of hair follicles, act as a reservoir of cytokines and regulate hair growth by affecting other cells in hair follicles [24]. The number of DPCs is important for maintaining the anagen phase, hair size and hair shape [25]. Once the DPCs’ numbers decrease, a destructive hair cycle and even alopecia will occur [26]. Therefore, proliferation ability becomes the most widely tested marker of DPCs’ activity [27]. Here, MTS-based assays, colony forming assays and Ki67 staining assays all showed that Hordenine had a positive, dose-dependent effect on DPCs’ proliferation. ALP and Versican are two well-established markers of DPCs’ inductivity [28,29]. Q-PCR and Western blot assays showed that Hordenine markedly upregulated the expression of ALP and Versican in DPCs. These data elucidate the effectiveness of Hordenine in increasing the proliferation and activity of DPCs.

It is reported that the maintenance of DPC-inductive properties is necessary for hair-follicle regeneration and growth of the hair shaft [30]. Indeed, local application of Hordenine in mouse dorsal skin accelerated anagen entry, indicating that Hordenine has the ability of promoting hair regrowth. Consistently, in a model of an ex vivo organ culture of hair follicles from mouse, Hordenine treatments significantly promoted hair shaft elongation. Therefore, we conclude that Hordenine has a potential role in promoting hair growth by increasing the activities of DPCs.

The Wnt signaling pathways have been demonstrated to play a significant role in the promotion of hair growth and hair follicle morphogenesis [31]. Moreover, this effect of the Wnt signaling pathways is mainly mediated by their action on the maintenance of DPCs’ activities [32]. Interestingly, in the present study, Hordenine treatments led to both the marked nuclear entrance of β-catenin and the increase in its downstream gene expression in DPCs. These findings were also confirmed in a mouse model. Furthermore, the increased proliferation and hair growth by Hordenine treatments can be fully reversed by an inhibitor of Wnt/β-catenin signaling, FH535. These findings indicate that Hordenine promotes DPCs’ proliferation and hair growth depending on activating the Wnt/β-catenin signaling pathway. Interestingly, we found that the Wnt3a expression in DPCs were markedly increased in a dose-dependent manner after Hordenine treatments. It is well-known that the Wnt3a ligand is a canonical Wnt family-member and can induce the accumulation of β-catenin, thereby activating the canonical Wnt signaling pathway. Here, we thus assumed that Hordenine can activate the Wnt/β-catenin signaling pathway by upregulating the expression of Wnt ligands, which need further investigation in the future. A previous study reported that Pilomatrixoma, a benign hair follicle-derived tumor, develops based on a post-zygotic mis-sense mutation (D32Y) of the gene encoding beta-catenin and the subsequent nuclear increase in beta-catenin protein [33]. In this study, we did not observe the formation of pilomatrixoma in the mice treated with Hordenine. The reason may be that Hordenine-induced activation of the Wnt signaling pathway is not strong enough to cause pilomatrixoma formation, which also needs further verification in the future.

Previous studies have already demonstrated that Hordenine can inhibit inflammatory reactions via inhibiting NF-κB, AKT and MAPK signaling pathways [34]. The inflammatory cells infiltrate into the hair follicles, which can result in alopecia-like alopecia areata [35]. Meanwhile, it is known that patients with androgenetic alopecia often undergo hair follicle miniaturization due to the inactive DPCs [36]. Thus, the effects of Hordenine on alopecia areata and androgenetic alopecia will be investigated in the future.

In conclusion, the results of the present study provide the first evidence that Hordenine effectively promoted hair growth and regeneration by activating the Wnt/β-catenin signaling pathway. Hordenine exerted a significant stimulatory effect on increasing the activity of DPCs and accelerating the onset of anagen. Moreover, Hordenine is the active ingredient in germinated barley seeds, which has the merits of resource abundance and lower toxicity. Therefore, our findings suggested that Hordenine may be developed as an effective candidate to prevent and treat alopecia in the future.

## Data Availability

No new data were created or analyzed in this study. Data sharing is not applicable to this article.

## Supplementary Materials

The following supporting information can be downloaded at: https://www.mdpi.com/article/10.3390/nu15030694/s1, Figure S1. Hordenine’s toxicity to epidermal and dermal cells; Figure S2. The increased activity of DPCs by Hordenine is reversed by FH535.
