# Supplementary material for: Hordenine Activated Dermal Papilla Cells and Promoted Hair Regrowth by Activating Wnt Signaling Pathway
Source: Nutrients. 2023 Jan 30;15(3):694. doi: 10.3390/nu15030694 (PMC9921158; doi:10.3390/nu15030694)
Supplement: Supplementary file 1 [file nutrients-15-00694-s001.zip › Supplemental Information.pdf]

Hordenine activated dermal papilla cells and promoted hair regrowth by activating Wnt Signaling pathway

Caibing Wang[1], Kai Zang[2], Zexin Tang[1], Ting Yang[1], Xiyun Ye[1]*, Yongyan Dang[2]*

1 Institute of Biomedical Sciences, East China Normal University, Shanghai 200241, China

2 Shanghai Key Laboratory of Regulatory Biology , School of Life Science, East China Normal University, Shanghai 200241, China; 51201300103@stu.ecnu.edu.cn

* Correspondence: yydang@bio.ecnu.edu.cn (Y.D.); xyye@bio.ecnu.edu.cn (X.Y.); Tel.: +86-21-5434-5482 (X.Y.)

Supplemental Figures and Legends

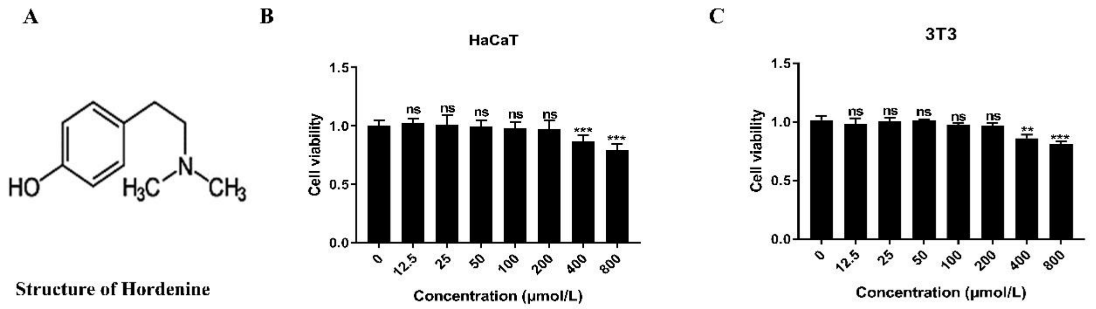

Supplementary figure 1. Hordenine's toxicity to epidermal and dermal cells. (A) Hordenine's structure. (B) Hordenine's toxicity to HaCaT cells and (C) 3T3 cells. HaCaT cells or 3T3 cells were treated with Hordenine range of 0-800 μM/L. Data are expressed as means ± SD. * $P < 0.05$, ** $P < 0.01$, *** $P < 0.001$, Hordenine versus control.

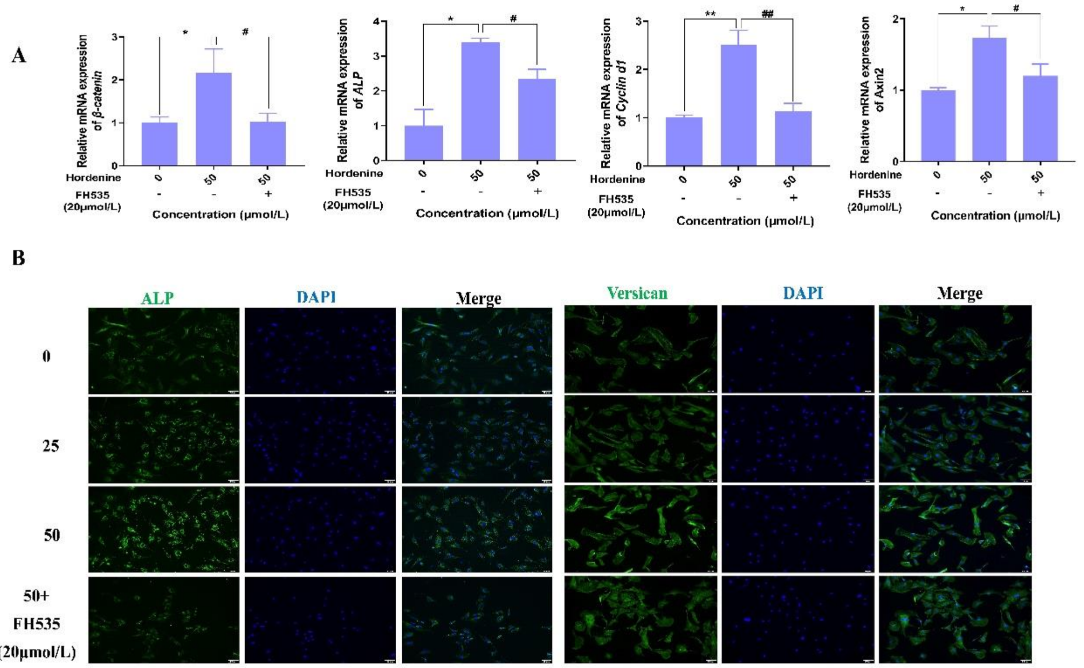

Supplementary figure 2. The increased activity of DPCs by Hordenine is reversed by FH535. (A) The mRNA expression of Wnt/β-catenin signaling pathway factors, *β-catenin, Lef-1*, *Cyclin d1* and *Axin2* were examined by quantitative RT-PCR. (B) Representative immunofluorescence images of ALP and Versican in DPCs treated with Hordenine in the presence or absence of FH535 (20 μmol/L) for 24 hours. (Magnification, ×200; bars = 50 μm). Data are expressed as means ± SD. * $P < 0.05$, ** $P < 0.01$, *** $P < 0.001$, Hordenine versus control; # $P<0.05$, ##$P<0.01$, Hordenine versus Hordenine plus FH535.
